# Implementation factors of tuberculosis control program in primary healthcare settings in China: a mixed-methods using the Consolidated Framework for Implementation Research framework

**DOI:** 10.1186/s40249-024-01222-3

**Published:** 2024-07-08

**Authors:** Jiani Zhou, Quan Yuan, Qingning Huang, Qingya Wang, Hexiang Huang, Wei Chen, Geng Wang, Shili Liu, Ting Zhang, Xi Zhao, Ying Li

**Affiliations:** 1 Department of Social Medicine and Health Service Management, Army Medical University (Third Military Medical University), Shapingba District, Chongqing Municipality, China; 2 Chongqing Tuberculosis Prevention and Control Institute, Chongqing Municipality, China; 3 Institute of Tuberculosis Prevention and Control, Center for Disease Control and Prevention, Guiyang, Guizhou Province China; 4 Guiyang Center for Disease Control and Prevention, Guiyang, Guizhou Province China

**Keywords:** Tuberculosis control, Primary setting, Implementation science, Barriers and enablers, Consolidated Framework for Implementation Research, China

## Abstract

**Background:**

Tuberculosis (TB) is a major cause of death worldwide, and Chinese TB burden ranked the second globally. Chinese primary healthcare (PHC) sectors implement the TB Control Program (TCP) to improve active case finding, referral, treatment adherence, and health education. This study aimed to identify barriers and enablers of TCP implementation in high TB burden regions of West China.

**Methods:**

We conducted a representative study using mixed-methods in 28 counties or districts in Chongqing Municipality and Guizhou Province of West China from October 2021 to May 2022. Questionnaire surveys and semi-structured in-depth interviews were conducted with 2720 TB healthcare workers (HCWs) and 20 interviewees in PHC sectors. Descriptive statistical analysis was used to investigate TB HCWs’ characteristics, and path analysis model was utilized to analyze the impact of associated factors on TCP implementation. Thematic framework analysis was developed with the guide of the adapted Consolidated Framework for Implementation Research (CFIR) on factors of TCP implementation.

**Results:**

This study found that 84.6% and 94.1% of community and village HCWs had low professional titles. Based on the results of multiple regression analysis and correlation analysis, lower TB core knowledge scores (-0.09) were identified as barriers for TCP implementation in community PHC sectors, and low working satisfaction (-0.17) and low working willingness (-0.10) are barriers for TPC implementation in village PHC sectors. The results of in-depth interviews reported barriers in all domains and enablers in four domains of CFIR. There were identified 19 CFIR constructs associated with TCP implementation, including 22 barriers such as HCWs’ heavy workload, and 12 enablers such as HCWs’ passion towards TCP planning.

**Conclusions:**

With the guide of the CFIR framework, complex factors (barriers and enablers) of TCP implementation in PHC sectors of West China were explored, which provided important evidences to promote TB program in high TB burden regions. Further implementation studies to translate those factors into implementation strategies are urgent needed.

**Graphical Abstract:**

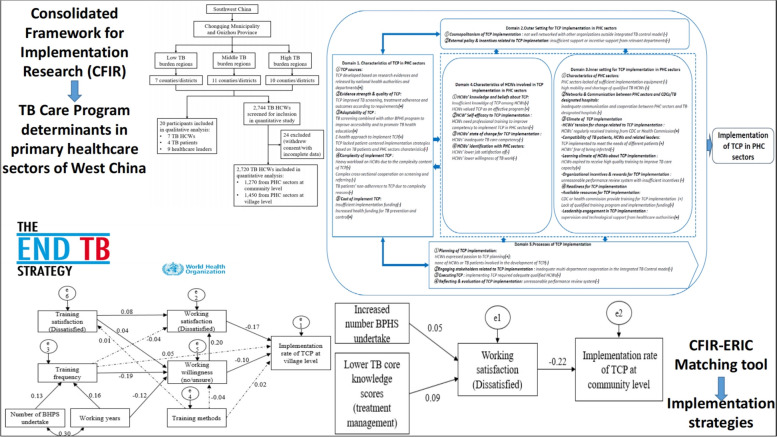

**Supplementary Information:**

The online version contains supplementary material available at 10.1186/s40249-024-01222-3.

## Background

Until coronavirus disease 2019 (COVID-19) pandemic, tuberculosis (TB) was the leading cause of death from a single infectious agent that seriously threatened public health [1]. According to the *2023 Global Tuberculosis Report* released by the World Health Organization (WHO), there were 10.6 million new TB cases worldwide, with an incidence rate of 134 per 100,000 populations compared with 2020 [1]. The COVID-19 pandemic had a devastating impact on the diagnosis, treatment, and burden of TB. The number of newly diagnosed TB cases decreased significantly, which indicated an increase in undiagnosed and untreated TB patients and an increase in TB deaths and transmission [1]. Among the 30 high-burden TB countries, China ranked the third in the number of drug-susceptible TB (DS-TB) cases, accounting for 7.1% of global DS-TB cases; and the fourth in the number of multidrug-resistant/rifampicin-resistant TB (MDR/RR-TB) cases, accounting for 7.3% of global MDR/RR-TB cases [1].

WHO formulated *the End TB Strategy*. It emphasized the role and importance of patient-centered and community-based care in TB control, as the length of TB treatment was prolonged with at least six months to two years combined with a high risk of drug side-effect and financial burden [2]. The Chinese government has made a strong commitment to end TB, and has implemented the Integrated TB Control model in most regions since 2012 [3, 4]. This model integrated the centers for disease control (CDCs), TB designated hospitals, and primary healthcare (PHC) sectors to provide high-quality TB care [4, 5]. Under the Integrated TB Control model, PHC sectors play its key role to provide free community-based TB Care Program (TCP) for TB patients, which covered actively case screening and referring, suspect case tracking, treatment management, and TB health education [6]. In China, community-based TCP was mainly delivered by TB healthcare workers (HCWs) in PHC sectors, which consisted of -[7]. All TCP programs are free for TB patients, because they are covered by the national Basic Public Health Service (BPHS) which are financed by the government [8]. Many previous studies have revealed that TCP provided by PHC sectors had positive effects on TB patients’ regular medication intake, service satisfaction, treatment outcomes, TB health literacy, and mental wellbeing [9–12]. However, the implementation outcomes of TCP vary greatly across China [12]. Even in the economically developed central and eastern regions, TCP implementation faces problems such as poor treatment management and TB health education [13]. In West China where the TB epidemic is more serious, the health service system is more backward, and the economy is less developed, the implementation of TCP is not as effective as expected. For example, more than 50% of TB patients had not been identified and referred by PHC sectors, and nearly two-thirds of family members of TB patients had never received close contact TB screening in Chongqing and Guizhou [5, 14, 15]; Only 17% DS-TB and 39.6% DR-TB patients received standardized management respectively, about 40% patients were unwilling to accept treatment management, and some patients even refused TCP provided by HCWs in Chongqing [14]. Only 12.2% TB patients had TB knowledge, which was far lower than the national requirement of 85%, and more than 50% of MDR-TB patients had not received TB health education from PHC sectors in West China [16, 17].

The implementation of evidence-based interventions often fails due to the reality that contextual factors are active and dynamic forces, both supporting and hindering implementation efforts in the real world [18]. If evidence-based effective programs/interventions were deployed incorrectly (implementation failure), the programs/interventions cannot achieve successful outcomes (treatment outcome or service outcome) [19]. Therefore, it is essential to understand the factors that impede and facilitate the implementation process of programs/interventions, in order to maximize the outcomes [20]. However, to the best of our knowledge, there were few studies to systematically explore the factors of TCP implementation in PHC sectors of West China where the TB burden was the heaviest [21]. The Consolidated Framework for Implementation Research (CFIR), the most widely used frameworks in implementation science, provides a comprehensive meta-theoretical framework to help unlock the complexities of healthcare implementation, by taking into account all the interacting elements in the implementation process [22]. The CFIR is composed of 5 major domains (intervention characteristics, outer setting, inner setting, characteristics of the individuals involved, and the process of implementation) and 37 constructs (8 constructs under the intervention characteristics, 4 constructs under outer setting, 12 constructs under inner setting, 5 constructs under individual characteristics, and 8 constructs under implementation process) [23]. Therefore, this study aims to investigate factors (barriers and enablers) of TCP implementation in West China with the guide of CFIR. Since TB is a global health problem, the exploration on barriers and enablers of TCP implementation in West China could contribute to the global experience of TB prevention and control, by paying attention to factors of TB intervention implementation in different contexts.

## Methods

### Study design and settings

**Fig. 1:**
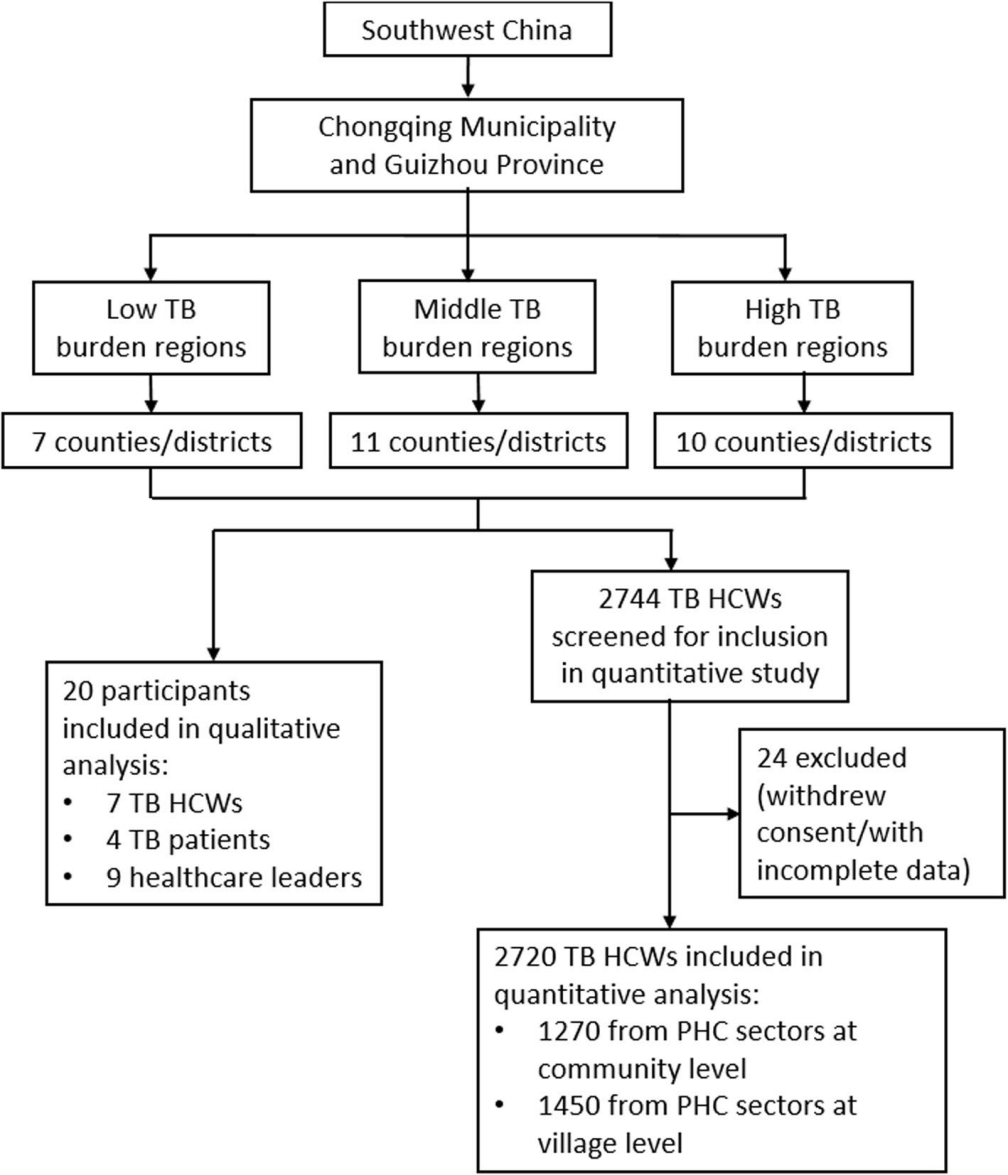
Study flowchart of mixed-study in Chongqing and Guizhou. *TB* Tuberculosis; *HCW* Healthcare worker; *PHC* Primary healthcare

We used a mixed-method approach to conduct questionnaire surveys and semi-structured in-depth interviews from October 2021 to May 2022. A multistage stratified sampling was utilized to select PHC sectors in Chongqing Municipality and Guizhou Province. Chongqing Municipality and Guizhou Province are two large provincial administrative regions located in West China. In 2021, the TB incidence rate in Chongqing was 61.7/100,000, higher than the national TB incidence rate of 45.5/100,000 [24]. The TB epidemic situation in Guizhou Province was even more serious, with a reported incidence rate of 102.51/100,000 in 2019, ranking after Xinjiang and Tibet [25]. Firstly, we purposively selected Chongqing Municipality (representing areas with better socioeconomic status and lower TB burden) [26, 27] and Guizhou province (representing areas with lower socioeconomic status and higher TB burden) [27, 28] according to different economic development and TB burden levels. Secondly, all counties/districts were grouped into three levels (low, middle, and high) according to their local TB incidences. At least 6 counties/districts (3 from each study place) were randomly chosen from each incidence level to ensure regionally representative. Eventually, 28 counties/districts in West China were chosen in this study (Fig. 1). The Strengthening the Reporting of Observational Studies in Epidemiology (STROBE) checklist was used by the research team to assess the study design, analysis, and findings [29].

### Analytical framework

**Fig. 2:**
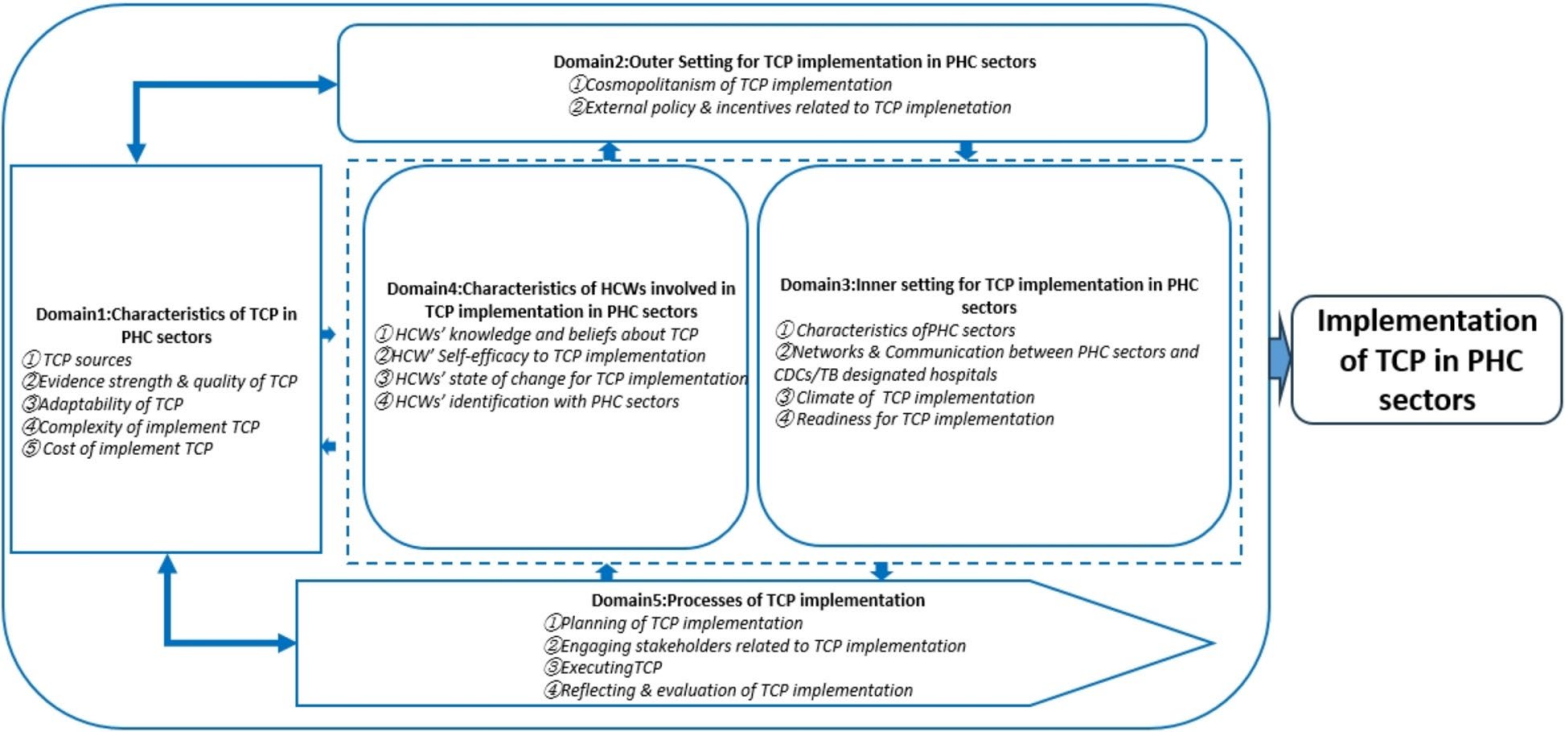
Adaptation of CFIR framework on factors of TCP implementation in PHC sectors. *CFIR* Consolidated Framework for Implementation Research; *TCP* Tuberculosis Control Program; *PHC* Primary healthcare; *HCWs* Healthcare workers

With the guide of CFIR, this study collected data on the factors of TCP implementation from the following 5 domains and 19 constructs: (1) Characteristics of TCP in PHC sectors (TCP sources, evidence strength & quality of TCP, adaptability of TCP, TB patient needs & resources, complexity of implement TCP and cost of implement TCP); (2) Outer setting of TCP implementation in PHC sectors (cosmopolitanism of TCP implementation, and external policy & incentives related to TCP implementation); (3) Inner settings of TCP implementation in PHC sectors (characteristics of PHC sectors, networks & communication between PHC sectors and CDCs/TB designated hospital, climate of TCP implementation, and readiness for TCP implementation); (4) Characteristics of individuals related to TCP implementation in PHC sectors (HCWs’ knowledge and beliefs about TCP, HCWs’ self-efficacy to TCP implementation, HCWs’ state of change for TCP implementation, and HCWs’ identification with PHC sectors); and (5) Process of TCP implementation in PHC sectors (planning of TCP implementation, engaging stakeholders related to TCP implementation, executing TCP and reflecting & evaluation of TCP implementation). The adaptation of CFIR framework on factors of TCP implementation in this study is illustrated in Fig. 2.

### Participants and data collection

#### Quantitative study

All TB HCWs in PHC sectors at community (community health centers/stations and township hospital centers) and village level (village clinics) who were responsible for TCP implementation from the selected 28 counties/districts were targeted in this survey. Those willing to participate were recruited with the assistance of local CDCs. Our research group designed a structured questionnaire based on CFIR, policies and regulations, and consulted with relevant experts. The questionnaire included: (1) demographic information, such as education, working region, and professional title; (2) training status, such as frequency and approach; (3) knowledge about TCP in PHC sectors (discovery, recommendation and tracking, patient treatment, treatment management, and health education), covering a total of 30 questions, one point for each question; and (4) the implementation status of TCP. The sample size was estimated using the formula for stratified sampling: $n = \frac{\sum {W}_{i}^{2}{S}_{i}^{2}/{w}_{i}}{V+\sum {W}_{i}{S}_{i}^{2}/N}$, where*, n* is the minimum desired sample size,* W*_*i*_ = *N*_*i*_*/N*, V = (δ/u_α/2_)^2^, and u_α/2_ = 1.96, corresponding to a 5% level of significance. The calculated minimum sample size for one provincial level study region was about 500. Therefore, the minimum sample size for this study was 1000, and the minimum sample size for each stratified level was 333. We randomly selected 10% of questionnaires for review and the compliance rate was over 95%. Questionnaires were collected through an online survey tool and distributed by the leaders of PHC sectors to HCWs who deliver the TCP program. We introduced the objectives and details of this study to potential participants in the online informed consent. Each completed questionnaire was checked and examined for quality control by trained investigators.

#### Qualitative study

The purposive sampling method was used to select participants for the qualitative study and the sample size was determined according to the point of data saturation [30]. Semi-structured in-depth interviews were conducted with TB HCWs, TB patients, leaders of the local CDCs and the local health commission. During the interviews, semi-structured topic guides were utilized, which were designed under the guide of CFIR from five domains of TCP. Interviews with HCWs, and TB patients emphasized individual characteristics, intervention characteristics and the implementation process, whereas interviews with the leaders of CDC and health commission centered on the outer setting, inner settings, and intervention characteristics. Recruitment was facilitated by faculties in the selected study districts who explained about the study and its objectives. All interviews were conducted in the local dialect by trained researchers and recorded digitally with the participants’ consent. Each interview lasted approximately 40–60 min.

### Data analysis

#### Quantitative analysis

Data were cleaned in Microsoft Excel. The double-checking method was used, and SPSS 22.0 (IBM, New York, USA) and AMOS 24.0 (IBM, New York, USA) were used for data analysis. Descriptive statistical analysis was used to investigate TB HCWs’ demographic, working characteristics, training situation, TB care knowledge, and TCP implementation status. Continuous variables with normal distribution were represented by mean and standard deviation (mean, SD); categorical variables were represented by number and percentage (*n*, %). A two-tailed probability level of *P-*value < 0.05 was considered statistical significance.

Path analysis model was utilized to analyze the impact of associated factors on TCP implementation. The path model diagram of the influence of various variables on the TCP implementation was constructed through multiple model fitting tests and modifications. The main indicators [31] of model evaluation of path analysis models were: (1) Overall model fitting test via the Chi-square value (*χ*^2^), and the better the fit of the hypothesis model to the actual observed data, and the corresponding test probability *P* > 0.05 indicates that the model fits well; *df* represents degrees of freedom, and the smaller the *χ*^2^/*df* value is, the better the fit of the hypothesis model to the observed data. Generally, it is believed that *χ*^2^/*df* value should be less than 3. (2) Incremental fit index which compares the fit of the hypothesis theoretical model to the baseline model to determine the fit of the model. Commonly used indicators include the standardized fitting index (Normed Fit Index, NFI), Relative Fit Index (RFI), Incremental Fit Index (IFI), non-standardized fit index (Tracker-Lewis Index, TLI) and Comparative Fit Index (CFI). The value of the incremental fit index is usually between 0 and 1, the closer it is to 1, the better the model fits, and the index should generally be greater than 0.9. (3) Root Mean Square Error of Approximation (RMSEA). The closer the RMSEA value is to 0, the better the model fit. Generally, if it is between 0.05–0.08, the model fit is good and has reasonable fit; if it is less than 0.05, the model fit is very good. In addition, if the absolute value of the path coefficient is less than 0.01, the effect is weak; if it is between 0.10–0.50, the effect is medium; if it is greater than 0.50, the effect is large. Weighted least squares method was used to fit and modify the model when the variables do not follow multivariate normal distribution.

#### Qualitative analysis

Data from interviews were transcribed from digital recordings into word documents manually, which were carefully reviewed for accuracy. Thematic Framework analysis was employed [32], following a five-step process [33, 34]. A theoretical framework was developed with the guide of the adapted CFIR framework on factors of TCP implementation. The names of all participants in the in-depth interviews were removed from the quotations in the results to maintain their anonymity. This study followed the Consolidated Criteria for Reporting Qualitative Research (COREQ) guideline.

## Results

### Characteristics of participants

In total, 2720 TB HCWs were included in the final analysis, 1270 from PHC sectors at community level and 1450 from PHC sectors at village level (Table 1). More than half of HCWs attended junior medical college in communities and technical secondary school or below in villages. The majority of both community and village HCWs had medical education backgrounds (89.8% and 84.1%, respectively). The income of community HCWs was higher than that of village HCWs. Moreover, most HCWs had low professional titles, 84.6% and 94.1% of community and village HCWs had non/junior titles respectively. A larger proportion of village HCWs were undertaking more than 5 BPHS programs compared with community HCWs (75.4% vs 29.1%), and a greater proportion of village HCWs had longer working years than community HCWs. As for working satisfaction and willingness, less than 50% of both community and village HCWs were satisfied with TCP implementation, and more community HCWs had low working willingness (40.0%) than village HCWs (25%). Eventually, 20 interviewees participated in the in-depth interviews. This included 7 TB HCWs (3 from community areas and 4 from village areas), 4 TB patients (2 with DS-TB and 2 with DR-TB), and 9 health care leaders (6 from CDC and 3 from the Health Commission). The demographic characteristics of the interviewed HCWs matched those of the surveyed HCWs. All four interviewed TB patients had completed treatment. Of the nine health care leaders interviewed, five held a deputy senior title, and all had at least three years of administrative working experience in TB-related fields.

**Table 1:** Demographic and working characteristics of TB HCWs in PHC sectors in West China, *n* (%)

**Characteristics**	**HCWs at community level**	**HCWs at village level**	*χ*^**2**^	***P***
Gender (*n* = 1270, 1450)			106.683	< 0.001
Female	452 (35.6)	803 (55.4)		
Male	818 (64.4)	647 (44.6)		
Age, years (*n* = 1270, 1450)			406.789	< 0.001
20–29	457 (36.0)	158 (10.9)		
30–39	399 (31.4)	291 (20.1)		
40–49	303 (23.9)	631 (43.5)		
> 49	111 (8.7)	370 (25.5)		
Education (*n* = 1270, 1450)			459.572	< 0.001
Technical secondary school or below	216 (17.0)	790 (54.5)		
Junior medical college	653 (51.4)	521 (35.9)		
Undergraduate college or above	401 (31.6)	139 (9.6)		
Medical education (*n* = 1270, 1450)			19.075	< 0.001
Yes	1140 (89.8)	1219 (84.1)		
No	130 (10.2)	231 (15.9)		
Major (*n* = 1115, 1219)			343.914	< 0.001
Clinical Medicine	365 (28.7)	300 (20.7)		
General Medicine	79 (6.2)	386 (26.6)		
Public Health	117 (9.2)	135 (9.3)		
Chinese traditional medicine	92 (7.2)	167 (11.5)		
Nursing	313 (24.6)	96 (6.6)		
Others	149 (11.7)	135 (9.3)		
Monthly income, CNY (*n* = 1270, 1447)			662.432	< 0.001
< 2500	255 (20.1)	961 (66.3)		
2500–3500	344 (27.1)	287 (19.8)		
3500–4500	345 (27.2)	104 (7.2)		
> 4500	326 (25.7)	95 (6.6)		
Professional title (*n* = 1270, 1450)			215.879	< 0.001
None	465 (36.6)	928 (64)		
Junior	610 (48.0)	436 (30.1)		
Intermediate	159 (12.5)	76 (5.2)		
Deputy senior/Senior	36 (2.8)	10 (0.7)		
Number of BPHS undertake (*n* = 1270, 1450)			97.044	< 0.001
≤ 2	385 (30.3)	213 (14.7)		
3–4	515 (40.6)	143 (9.9)		
≥ 5	370 (29.1)	1094 (75.4)		
Working years in PHC sectors (*n* = 1214, 1450)			86.058	< 0.001
≤ 2	498 (39.2)	374 (25.8)		
3–4	230 (18.1)	183 (12.6)		
≥ 5	542 (42.7)	892 (61.5)		
Working satisfaction to implement TCP in PHC sectors (*n* = 1270, 1450)			32.831	< 0.001
Satisfied	552 (43.5)	696 (48.0)		
Normal	482 (38.0)	406 (28.0)		
Dissatisfied	236 (18.6)	348 (24.0)		
Willingness to implement TCP in PHC sectors (*n* = 1214, 1450)			68.459	< 0.001
Yes	728 (60.0)	1087 (75.0)		
No/unsure	486 (40.0)	363 (25.0)		

Missing data were excluded from the analysis, and the final sample size for TB HCWs at the community and village levels is indicated after the name of each variable. *TB* Tuberculosis, *HCW* Healthcare worker, *CNY* Chinese Yuan, *BPHS* Basic public healthcare services, Under *Major* variable, *Others* include Combination of Chinese Medicine and Western Medicine, Rural Medicine, and Community Medicine

### Training and TB knowledge of TCP among HCWs in PHC sectors

Training for TCP implementation was carried out among HCWs in PHC sectors (Table 2). The majority of both community HCWs (48.0%) and village HCWs (34.6%) had received training less than once half-year. They mainly obtained training through offline lectures/meetings or a combination of offline and online methods. More than 80% community and village HCWs preferred to have training content on TB knowledge, skills of treatment management for TB patients, referring suspicious TB patients, health education on TB knowledge, and close contacts screening and tracing. Though around 90% HCWs in PHC sectors were satisfied with training, knowledge about TCP implementation among both community and village HCWs were low (Table 2), with an overall score of 12.945 and 12.185 respectively, less than half of the total score of 30.

**Table 2:** Training and knowledge about TCP among TB HCWs in PHC sectors in West China, *n* (%)

**Variable**	**HCWs at community level**	**HCWs at village level**		
**Training of TCP**			*χ*^**2**^	***P***
**Training frequency**			86.058	< 0.001
≤ 1/half-year	583 (48.0)	502 (34.6)		
2/half-year	418 (34.4)	479 (33.0)		
≥ 3/half-year	213 (17.5)	469 (32.3)		
**Training methods**			116.583	< 0.001
Offline lecture/meeting	530 (45.7)	690 (55.2)		
Online lecture/meeting	257 (25.2)	85 (6.8)		
Mixed	372 (32.1)	474 (38.0)		
**Preferred training methods**			6.589	< 0.001
Offline lecture/meeting	1034 (81.4)	1166 (80.4)		
Online lecture/meeting	709 (55.8)	914 (63.0)		
Live guide	945 (74.4)	1209 (83.4)		
Observing training	794 (62.3)	925 (63.8)		
**Preferred training content**			2.347	< 0.001
TB core knowledge	1108 (87.2)	1309 (94.4)		
Skill of treatment management for TB patients	1129 (88.9)	1267 (91.4)		
Skill of referring suspicious TB patients	1074 (84.6)	1200 (86.6)		
Skill of health education on TB knowledge	1061 (83.5)	1175 (84.8)		
Skill of close contacts screening and tracing	1115 (87.8)	1210 (87.3)		
**Training satisfaction**			119.936	< 0.001
Satisfied	975 (89.9)	1211 (99.8)		
Dissatisfied	110 (10.1)	3 (0.2)		
**TB HCWs’ knowledge about TCP, Mean (SD)**				
1. Screening, referral, and tracking (including 8 questions)	3.907 (1.612)	3.542 (1.528)		< 0.001*
2. Anti-TB treatment regime and policy (including 8 questions)	2.514 (1.315)	2.003 (1.241)		< 0.001*
3. Treatment management (including 8 questions)	3.671 (1.289)	3.574 (1.330)		0.172
4. Health education (including 6 questions)	3.106 (0.840)	3.121 (0.882)		0.526
Overall TB knowledge score	12.945 (3.291)	12.185 (3.198)		< 0.001*

*TB* Tuberculosis, *TCP* TB control program, *HCW* Healthcare worker, *PHC* Primary healthcare, * indicates *P* < 0.05

### TCP implementation in PHC sectors and associated factors

**Fig. 3:**
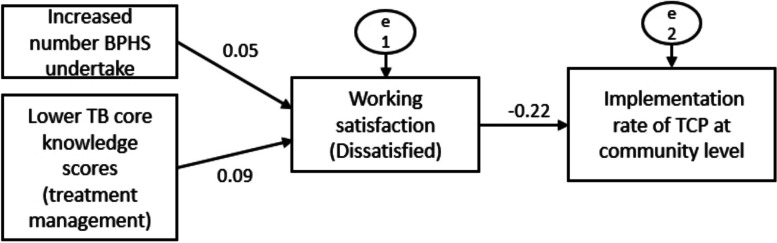
Path model of TCP implementation in PHC sectors at community level. Note: the solid line represents the path coefficient *P* < 0.05, and the dashed line represents the path coefficient *P* ≥ 0.05. *BPHS* Basic public health service; *TB* Tuberculosis; *TCP* Tuberculosis Control Program; *PHC* Primary healthcare; *HCWs* Healthcare workers

Despite the higher implementation rate of TCP in urban regions (84.4%) than in rural areas (79.3%), the implementation status of 10 categories in TCP was similar in both settings (Table 3). Six categories showed a relatively better implementation status: TB prevention services for the publics, TB care services during the first-time follow-up visit for patients, health education for TB patients, follow-up evaluation and intervention for TB patients, case closed assessment for TB patients, and TB prevention services for patients’ family members. Conversely, four categories had a relatively low implementation rate: TB prevention services for high-risk populations, referred suspicious TB patients, treatment management for TB patients, and other services for TB patients. At the community level, regarding the path analysis of TCP implementation, 12 variables were determined after controlling demographics variables, including number of BPHS undertake, training frequency, working satisfaction, working years, training methods, training satisfaction, and TB core knowledge total scores. The fitting result of the path model concerning the implementation of TCP at both community and village levels indicated a good degree of fit (Table 4). The fitting result of the model is: *χ*^2^ = 5.068, *P* = 0.167, *df* = 3, *χ*^2^/*df* = 1.689 < 3, NFI = 0.936 > 0.9, RFI = 0.901 > 0.9, IFI = 0.973 > 0.9, TLI = 0.944 > 0.9, CFI = 0.972 > 0.9, RMSEA = 0.024 < 0.05. As shown in Fig. 3, low job satisfaction (-0.22) is a barrier for the implementation of TCP; increasing number BPHS undertake (-0.05) and lower TB core knowledge scores (treatment management) (-0.09) are indirect barriers for the implementation of TCP at community level.

**Table 3:** Implementation rate of TCP among TB HCWs in urban and rural settings in West China, *n* (%)

**Items of TCP**	**Urban HCWs** **(*n* = 1214)**	**Rural HCWs** **(*n* = 1450)**	*χ*^**2**^	***P***
1. TB prevention services for the public (including 2 specific TB care services)	89.8	82.2	4.364	0.886
2. TB prevention services for high-risk populations (including 5 specific TB care services)	79.2	73.1		
3. Referred suspicious TB patients (including 10 specific TB care services)	79.2	73.1		
4. TB care services during the first-time follow-up visit for patients (including 12 specific TB care services)	88.1	88.1		
5. Health education for TB patients (including 10 specific TB care services)	91.0	84.6		
6. Treatment management for TB patients (including 5 specific TB care services)	76.7	73.5		
7. Follow-up evaluation and intervention for TB patients (including 18 specific TB care services)	87.5	84.1		
8. Close contacts screening (including 3 specific TB care services)	91.5	85.1		
9. Other services for TB patients (including 9 specific TB care services)	71.4	69.8		
10. TB prevention services for patients’ family members (including 5 specific TB care services)	89.5	82.5		

*TB* Tuberculosis, *TCP* TB control program, *HCW* Healthcare worker, *PHC* Primary healthcare

**Table 4:** Fitting results of path analysis in TCP implementation at community and village levels in West China

**Level**	${{\varvec{\chi}}}^{2}$	***P***	${\varvec{d}}{\varvec{f}}$	${{\varvec{\chi}}}^{2}/{\varvec{d}}{\varvec{f}}$	**NFI**	**RFI**	**IFI**	**TLI**	**RMSEA**
Community	5.068	5.068	5.068	5.068	5.068	5.068	5.068	5.068	5.068
Village	15.570	15.570	15.570	15.570	15.570	15.570	15.570	15.570	15.570

*χ*^2^ Chi-square value, *P* corresponding test probability, *df* degrees of freedom, *NFI* normed fit index, *RFI* relative fit index, *IFI* incremental fit index, *TLI* tracker-lewis index, *RMSEA* Root mean square error of approximation

**Fig. 4:**
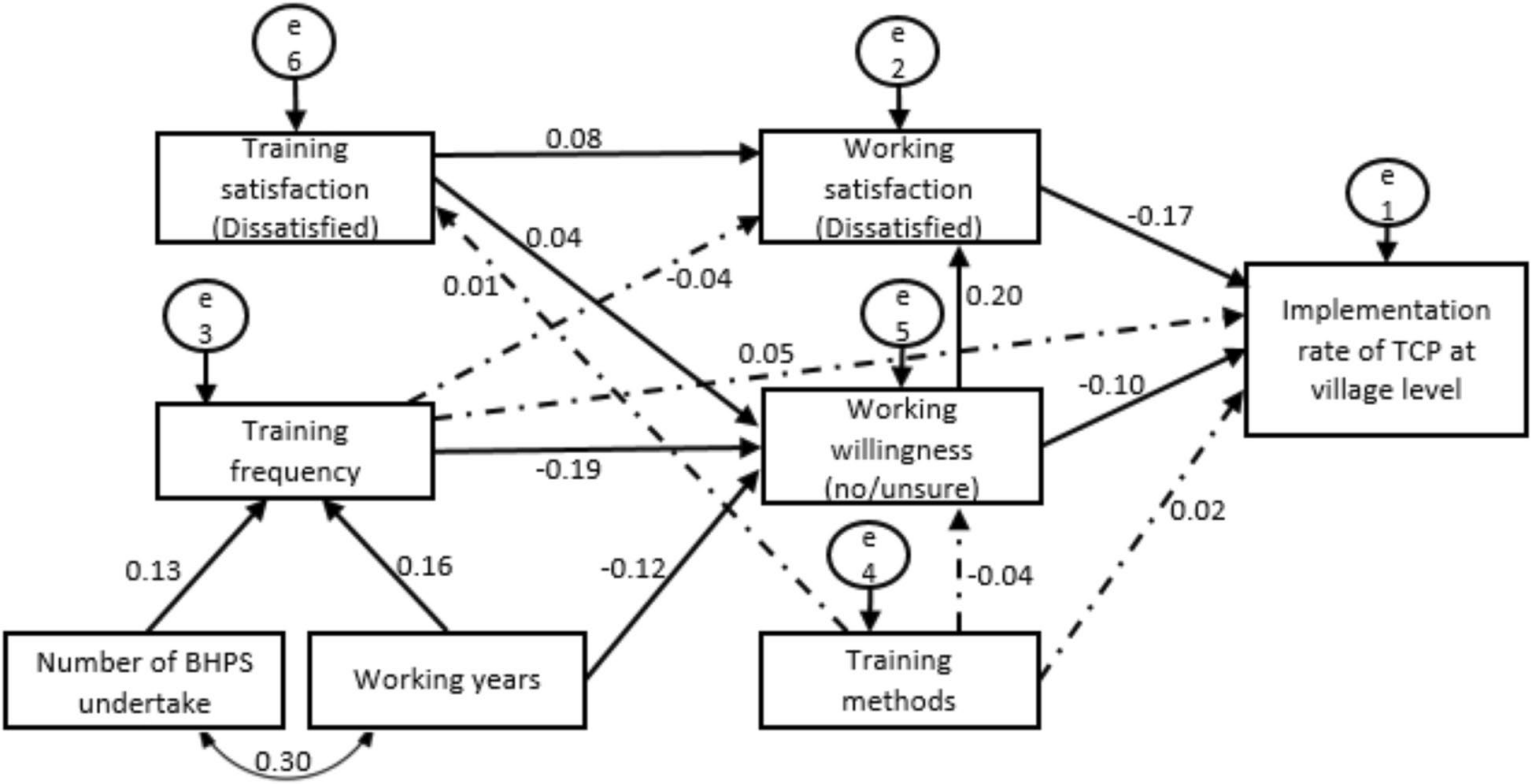
Path model of TCP implementation in PHC sectors at village level. Note: the solid line represents the path coefficient *P* < 0.05, and the dashed line represents the path coefficient *P* ≥ 0.05. *TCP* Tuberculosis Control Program; *PHC* Primary healthcare

At village level, after controlling for demographic variables, based on the results of multiple regression analysis and correlation analysis, 14 variables were identified in the path analysis of village TCP implementation, including number of BHPS undertake, working years, training frequency, training methods, training satisfaction, working satisfaction, working willingness, and TB core knowledge scores. The fitting result of the path model is: *χ*^2^ = 15.570, indicating that the model is well adapted to the actual observation data, *P* = 0.212 > 0.05 indicating that the model has good adaptability, *df* = 12, *χ*^2^/*df* = 1.297 < 3, NFI = 0.967 > 0.9, RFI = 0.923 > 0.9, IFI = 0.992 > 0.9, TLI = 0.981 > 0.9, CFI = 0.992 > 0.9, RMSEA = 0.014 < 0.05. As shown in Fig. 4, in terms of direct effects, low working satisfaction (-0.17) and low working willingness (-0.10) are barriers for the implementation of TCP at village level. As for the indirect effects, increased training frequency and working years are enablers for the implementation of TCP at village level, while low training satisfaction (dissatisfaction) is a barrier.

The path analysis indicated major barriers including low job satisfaction, TB core knowledge, working satisfaction and working willingness need urgent interventions to address.

### Qualitative results on factors of TCP implementation in PHC sectors

Factors of TCP implementation in PHC sectors included the following five domains (Table 5).

**Table 5:** Barriers and enablers in TCP implementation identified in interviews using the CFIR framework in West China

CFIR framework	Barriers and enablers
**Domain 1: Intervention characteristics–-TCP program**	
*Intervention source*	**Enablers:** 1. All HCWs and leaders stated TCP was developed by the national health authorities together with multi-related national departments such as education and financing, which required all regions to implement across China *I simply followed the instructions given to me by the National Health Commission.” –HCW*
*Evidence Strength & Quality*	**Enablers:** 1. Most HCWs and leaders mentioned that TCP significantly improved the TB screening, treatment adherence and outcomes according to instructions in BPHS *“We nowadays could find more suspicious TB cases.” –HCW*
*Adaptability*	**Enablers:** 1. Most PHC sectors combined TB screening with delivering other BPHS program to improve accessibility, and tried diverse activities to promote TB health education *“We utilize the World TB Day to conduct health education to the public and provide regular education for students.” –CDC leader* 2. Some HCWs tried e-health approach to implement TCP *“At the very least, we can alert younger patients through mobile phone software or something they more likely to accept.” –HCW* **Barriers:** 1. Both TB patients and HCWs reported TCP lacked patient-centered approaches to deliver *“I received calls (from HCW) during the first two months of my treatment… I am not preferring this approach.” –DS-TB patient*
*Complexity*	**Barriers:** 1. All HCWs stated TCP placed a heavy workload on them due to the complexity content of TCP (including 10 items) 2. Screening TB for high-risk population and referring suspicious/TB patients need widely cross-sectional cooperation, but the cooperation is difficult *“The governments require relevant departments, such as finance, education, civil affairs and social security, to participate in TB prevention, but most of them have little support and enthusiasm in participation.” –HCW* 3. HCWs reflected TB patients’ non-adherence to TCP due to complex reasons, such as TB patients lacked of beliefs and understandings of TCP in PHC sectors comparing with clinic treatment, had severe side effects of anti-TB treatment, financial difficulties, lived far away or were migrants, and had low health literacy *“It is unhelpful to call me every day (to remind me to take anti-TB drugs) …I really don’t like this way.” –DS-TB patient* *“They (TB patients) just say I don’t have money and never answer the phone call from me.” –HCW* *“Liver protection drugs and CT were not covered by medical insurance for TB. I don’t’ have money.” –DS-TB patient*
*Cost*	**Barriers:** 1. Leaders and HCWs reported most PHCs faced insufficient implementation funding for TCP *“Since TB treatment management has been included in BPHS, subsidies as a whole were given for BPHS and there was no special found for TCP.” –CDC leader* **Enablers:** 1. Leaders reported the government had increased health funding for TB prevention and control such as expending medical insurance coverage *“The anti-TB drugs for diagnosed patients are basically free, and the funding for TB health promotion is keep rising.” –Health Commission leader*
**Domain 2: Outer Setting – factors external to the organization delivering TCP**	
*Cosmopolitanism*	**Barriers:** 1. HCWs and leaders reported that PHC sectors were not well networked with other organizations outside the integrated TB control model, such as low support from private clinics or the departments of education, public security, civil affair, and finance during TB suspects or patients trace and TB health education *“Private clinics, hospitals and some pharmacies also withhold patients. They want to earn money for their hospital.” –HCW* *“It is hard to get support from social security department to contact with floating patients. And it is hard to recruit community volunteers for health education activities.” –HCW*
*External Policy & Incentives*	**Barriers:** 1. HCWs reported it was difficult to implement TCP in PHC sectors with insufficient support from relevant sectors outside the integrated TB control model, such as education, financial or public security departments, etc *“The governments require relevant sectors to participate in TB prevention, but most of them provide little support.” –HCW* *“It is hard to get support from social security department to contact with floating patients. And it is hard to recruit community volunteers for health education activities.” –HCW*
**Domain 3: Inner Setting—Inner Setting for TCP implementation**	
*Structural characteristics*	**Barriers:** 1. Most HCWs reported the PHC sectors lacked sufficient equipment for TCP implementation *“TB is an infectious disease; I don’t even have N95 masks when providing TCP” –HCW* 2. Leaders stated that the high turnover and shortage of qualified TB HCWs was found in most PHC sectors *“HCWs are very unstable, especially in rural areas. The low salaries can’t retain HCWs.” –CDC leader*
*Networks & Communication*	**Barriers:** 1. Inadequate communication and cooperation between PHC sectors and TB-designated hospitals influenced TCP delivery *“We don’t have a good communication channel with doctors from TB-designated hospitals. Patients don’t trust us.”–HCW* *“The doctor (from TB-designated hospital) never mentioned TCP to me.” –DS-TB patient*
*Implementation climate:* *-Tension for change* *-Compatibility* *-Organizational incentives & rewards* *-Learning climate*	**Barriers:** 1. Many HCWs mentioned the fear of being infected *“I feared being infected with TB.” –HCW* 2. Many HCWs mentioned inappropriate performance assessment with insufficient incentives largely influenced TB HCWs’ enthusiasm and willingness of TCP implementation *“The performance assessment is unreasonable. The more work you do, the more mistakes you could make.” –HCW* **Enablers:** 1. All PHC sectors regularly received training from CDC or Health Commission. All HCWs wish to receive high quality training to improve their TB care capacity 2. Some HCWs suggested that treatment management should be implemented in various ways to meet the needs of different patients *“I think that we can remind patients to take their medication through the internet. Although older people may be unfamiliar with phones, we often remind them through calls. At the very least, we can alert younger patients through mobile phone software or something they more likely to accept.” –HCW* 3. All HCWs wish to receive high quality training to improve their TB care capacity
Readiness for Implementation: -Leadership engagement -Available resources	**Barriers:** 1. HCWs and leaders reported insufficient funding for TCP implementation *“Funds for screening equipment, for health education, for training…These are still relatively lacking.” –CDC leader* 2. All HCWs stated though lots of training program, the current training did not satisfy HCWs’ actual needs to deliver TCP *“The training was focused on policies and guidelines related to TCP, and did not provide necessarily knowledge and skills in TCP delivery.” –HCW* **Enablers:** 1. Leaders in PHC sectors/CDC paid attention to TCP implementation, and CDC or health commission provided training for TCP implementation 2. Regular supervision and technological support from Health Commission and/or CDC *“We provide training on TCP at least twice per year, mainly around the latest guideline and implementation requirements.” –CDC leader*
**Domain 4: Individuals—characteristics of the individuals involved in TCP implementation**	
*Knowledge and Beliefs about Intervention*	**Enablers:** 1. All interviewees valued TCP as an effective program *“We (PHC sectors) nowadays could find more suspicious TB cases. We utilize other BHPS to actively screening people, particularly people with diabetes and some elders.” –HCW*
*Self-efficacy*	**Barriers:** 1. All interviewed HCWs and CDC leaders reported HCWs’ incompetency and hoped to receive more adequate training on TB care knowledge and other skills, such as communication skills to improve their TCP implementation outcomes *“Training often combined with other (BPHS) trainings…and I think this training is not effective.”–HCW*
*Individual State of Change*	**Barriers:** 1. Leaders and HCWs reported that HCWs’ inadequate professionalism on TB care impacted TCP delivery, especially in rural areas *“Some HCWs are not professional so TB patients don’t trust them, particularly the village doctors.” – CDC leader*
*Individual Identification with Organization*	**Barriers:** 1. All HCWs reported that non-patient-centered manners led to patients’ dissatisfaction and resistance to TCP, thus influencing the performance assessment of HCWs, diminishing their enthusiasm and willingness of TCP implementation *“I am not satisfied with this job. If there are other opportunities in the future, I will consider leaving this job.” –HCW*
**Domain 5: Process – processes of TCP implementation**	
*Planning*	**Barriers:** 1. None of the interviewed HCWs or TB patients had been involved in the development of TCP *“No one inquired about my opinion on the methods of providing TCP services.” –TB patient* *“Nobody consulted me on the development of the TCP program. I simply followed the instructions given to me.” –HCW* **Enablers:** 1. All of the interviewed HCWs expressed passion to participate in TCP planning *“I would be very happy if I could help with the TCP improvement a little. Frontline workers know about practice the best.” –HCW*
*Engaging*	**Barriers** 1. Most HCWs stated inadequate multi-department cooperation in the integrated TB control model (PHC sectors, CDC, and TB-designated hospital) *“It is hard to connect with TB designated hospitals’ doctors, since they are very busy.” –HCW*
*Executing*	**Barriers:** 1. Both interviewed HCWs and leaders highlighted that it was difficult to implement TCP according to the instructions in BPHS guideline *“…They (HCWs) just complete tasks and will not really care about the quality of TCP.” –CDC leader* *“I only received telephone calls from HCW in township hospital, once per month.” –TB patient*
*Reflecting & Evaluating*	**Barriers:** 1. Most HCWs reported that the current performance review system was unreasonable *“The assessment indicators are not scientific, and some are difficult to be completed. Sometimes it feels like I am trying to finish these indicators rather than really care about TCP for the patients.” –HCW*

*TB* Tuberculosis, *TCP* TB control program, *CFIR* Consolidated Framework for Implementation Research, *HCW* Healthcare worker, *PHC* Primary healthcare, *CDC* Center for Disease Control, *MDR/RR* Multidrug-resistant/rifampicin-resistant, *DS* Drug-susceptible

#### Domain 1: Characteristics of TCP in PHC sectors

This domain included 5 constructs: TCP sources, evidence strength & quality of TCP, adaptability of TCP, TB Patient needs & resources, complexity of implement TCP and cost of implement TCP. TCP was developed by the government in China, was evidence-based and cost-effective, significantly promoted TB control, and demonstrated a certain degree of adaptability, which are the key enablers. However, TCP lacked patient-centered delivery approaches and was weak in adaptability to community context, had complexity content and insufficient implementation funding, which were main barriers.

#### Domain 2: Outer setting for TCP implementation in PHC sectors

Two constructs mainly including cosmopolitanism of TCP implementation (network between PHC sectors and external organizations outside of the integrated TB control model) and external policy & incentives related to TCP implementation (outside of the integrated TB control model) were taken into the domain of outer setting for TCP implementation in PHC sectors. Interviews revealed that PHC sectors was not well networked with external organizations, such as insufficient support from private clinics or other sectors outside the integrated TB control model and private clinics retaining suspected TB patients without referring to TB-designated hospitals, which were barriers for TCP implementation. It was difficult to implement TCP in PHC sectors without incentive policy or support from education, financial or public security departments.

#### Domain 3: Inner setting for TCP implementation in PHC sectors

In this study, inner settings for TCP widely included characteristics of PHC sectors, networks & communication between PHC sectors and CDCs/TB designated hospital, climate of TCP implementation (HCWs’ tension for change related to TCP implementation, compatibility of TB patients, HCWs and related leaders and organizational incentives & rewards for TCP implementation) and readiness for TCP implementation (available resources for TCP implementation and leadership engagement in TCP implementation and access to information and knowledge). Barriers of inner setting for TCP implementation included the lack of sufficient equipment, funding, adequate training programs, adequate communication and cooperation with TB-designated hospitals, qualified TB HCWs, and scientific performance assessment with appropriate incentives. On the other hand, a high demand for high-quality training to strengthen HCWs’ TB care capacity, regular supervision and technological support in PHC sectors from superior healthcare departments were considered as key enablers in this domain.

#### Domain 4: Characteristics of HCWs involved in TCP implementation in PHC sectors

This study included HCWs’ knowledge and beliefs about TCP, HCWs’ self-efficacy to TCP implementation, HCWs’ state of change for TCP implementation and HCWs’ identification with PHC sectors as constructs in this domain. HCWs’ values of TCP as an effective program were enablers in this domain. TB patients’ lack of better understanding of TCP, insufficient awareness on TB and health literacy, severe side effects and financial difficulties of TB patients led to their dissatisfaction towards TCP implementation which could also had a negative impact on HCWs’ willingness of TB work, HCWs’ inadequate professional knowledge and skills in TCP implementation, were barriers to TCP implementation.

#### Domain 5: Processes of TCP implementation

Planning of TCP implementation (the development of TCP implementation strategies in PHC sectors under the guideline of BPHS), engaging stakeholders related to TCP implementation, executing TCP (according to the requirement instructions in the guideline of BPHS) and reflecting & evaluation of TCP implementation were main constructs in this domain. Results disclosed that HCWs’ and TB patients’ none involvement in planning of TCP implementation led to the inadequate understanding of their needs and local contexts, insufficient cooperation within the integrated TB control model, difficulties to implement TCP, and the inappropriate performance assessment were barriers in TCP implementation process. HCWs’ passions of participating in TCP planning were enablers in this domain.

### Identified factors of TCP implementation in PHC sectors from mixed-method results

**Fig. 5:**
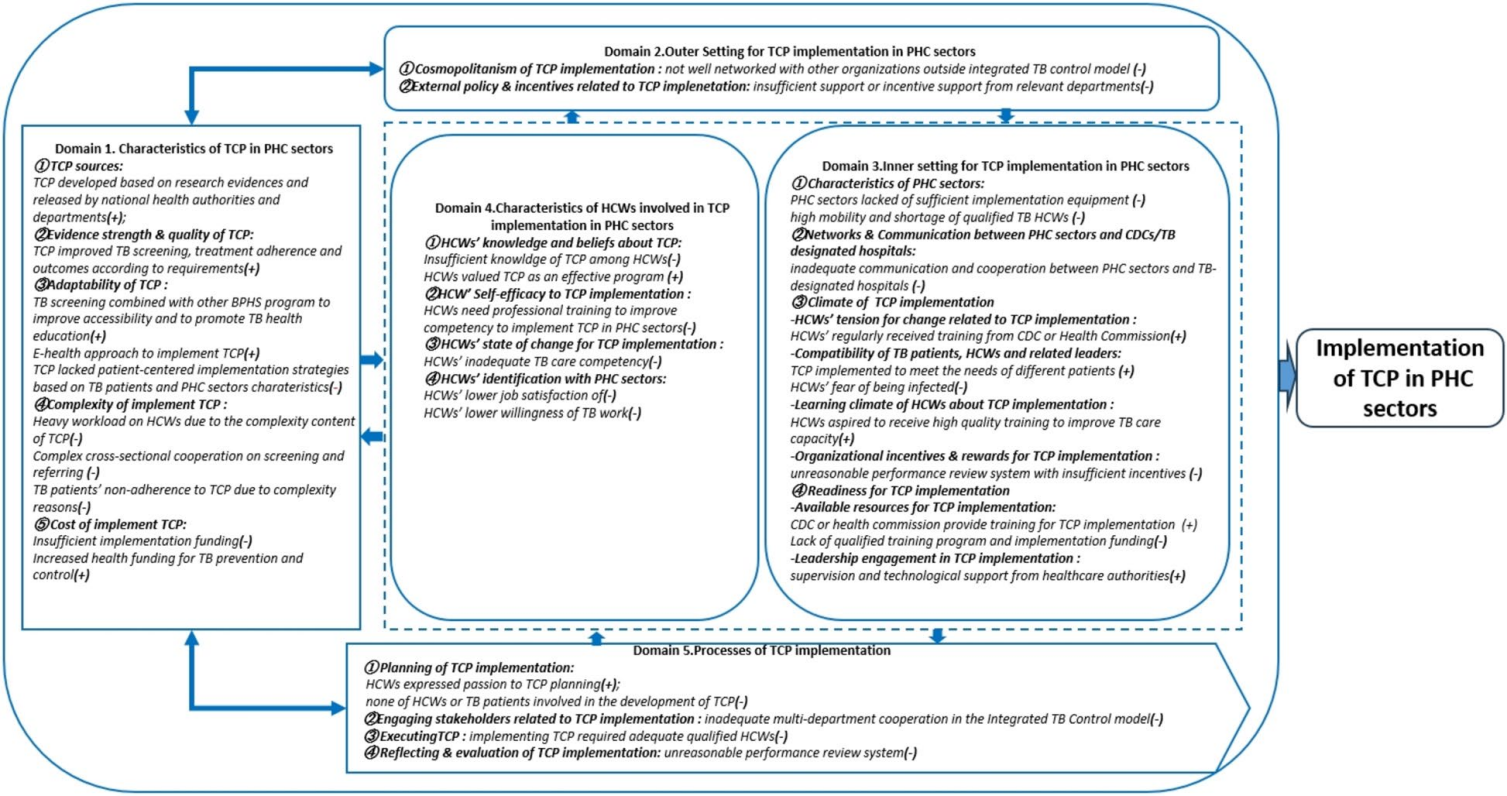
Barriers and enablers identified in TCP implementation using CFIR framework in PHC sectors. *CFIR* Consolidated Framework for Implementation Research; *CDC* Centers for Disease Control; *TCP* Tuberculosis Control Program; *PHC* Primary healthcare; *HCWs* Healthcare workers

Combining the results of both questionnaire surveys and in-depth interviews, we identified that 19 CFIR (Fig. 5) constructs on factors of the TCP implementation, covering 5 CFIR domains. Of these constructs, 22 were barriers, 12 were enablers.

Barriers existed widely in 5 domains: (1) Characteristics of TCP in PHC sectors: lack of patient-centered implementation strategies, heavy workload on HCWs, complex cross-sectional cooperation on screening and referring, TB patients’ non-adherence to TCP, and insufficient implementation funding; (2) Outer Setting for TCP implementation in PHC sectors: not well networked with other organizations and insufficient support or incentive from relevant departments; (3) Inner setting for TCP implementation in PHC sectors: lack of sufficient implementation equipment, high mobility and shortage of qualified TB HCWs, inadequate communication and cooperation between PHC sectors and TB-designated hospitals, HCWs’ fear of being infected, unreasonable performance review system with insufficient incentives and lack of qualified training program and implementation funding; (4) Characteristics of HCWs involved in TCP implementation in PHC sectors: the need of professional training, HCWs’ inadequate TB care competency, HCWs’ low job satisfaction of and low willingness of TB work; (5) Processes of TCP implementation: none of HCWs or TB patients involved in the development of TCP, inadequate multi-department cooperation, lack of adequate qualified HCWs and unreasonable performance review system.

Enablers arouse from 4 domains including TCP developed based on research evidences and released by national health authorities and departments, improved TB screening, treatment adherence and outcomes, TB screening combined with other BPHS program to improve accessibility and to promote TB health education, e-health approach and increased health funding for TB prevention and control, regular training from CDC or Health Commission, TCP meeting the needs of different patients, HCWs’ aspiration to receive high quality training, supervision and technological support from healthcare authorities, HCWs’ view of TCP as an effective program, and HCWs’ passion towards TCP planning.

## Discussion

TCP has been implemented across China, but its implementation has not achieved expected outcomes in western regions. Barriers to TCP implementation are complex which could arise at multiple levels including the TB patient level, the HCWs level, the organizational level and the policy level. However, the analysis of barriers lacked theoretical guidance and few research has paid attention to enablers in TCP implementation. CFIR can be used to guide diagnostic assessments of implementation contexts or implementation strategies to improve effectiveness of the intervention or program in a specific context [22]. To improve the TCP implementation in West China, barriers should be addressed and enablers should be strengthened accordingly. This study systematically identified the factors (barriers and enablers) of five domains in TCP implementation in West China with the guide of CFIR framework. Quantitative study showed low education levels and professional titles among HCWs, who also reported low satisfaction and willingness towards TB work. Quantitative study identified increased training frequency as enablers for TCP implementation, with a lack of sufficient training on TCP implementation knowledge as barriers. Qualitative results identified the lack of qualified HCWs and their low job satisfaction and willingness, and reflected although HCWs received regular training but they lacked qualified training program to improve their competency. Besides, qualitative results further pointed out more factors of TCP implementation such as the lack of patient-centered delivery approaches and insufficient implementation funding.

The first domain of the CFIR is characteristics of the intervention being implemented into a particular organization, which is often complex and multi-faceted with many interacting components [22]. WHO emphasized the importance of establishing patient-centered approaches to promote TB treatment adherence [35]. Consideration of patients’ needs and resources should be integrated into the design and implementation of health intervention programs. Studies in India and Pakistan found that patient-centered care was associated with improved adherence to TB treatment, better treatment outcomes and higher patient satisfaction [36, 37]. A case-control study in China also indicated that patient-centered care could help to improve the treatment rate and adherence and lessen the loss rate of MDR/RR-TB follow-up patients [38]. Previous studies conducted in Middle and West China found that the lack of patient-centered care was the significant barrier to deliver effective TCP in PHC sectors [5, 15, 39–41]. This study consistently found that TCP in PHC sectors lacked patient-centered perspective as a key barrier in West China, where patients were migrants or live far away from their hometowns, were elderly with poor health literacy, or had side effect to anti-TB drugs. Consistent with other studies [37, 40–42], this study found the complexity content of TCP program and insufficient implementation funding for TCP were barriers to TCP implementation in PHC sectors.

Successful implementation of health intervention requires considering contextual factors from both inner and outer settings [43, 44]. The outer setting includes the economic and social contexts which an organization resides [23], including cosmopolitanism (network between PHC sectors and external organizations outside of the integrated TB control model), and external policy and incentives outside of the integrated TB control model. Organizations that support and promote external boundary-spanning roles are more likely to implement new practices quickly [23]. But few studies analyzed barriers from PHC sectors and multi-sector cooperation in TCP implementation [45, 46]. This study identified PHC sectors were not well networked with other organizations outside the integrated TB control model, and few policies and incentives from other organizations supported TCP implementation in PHC sectors. The social factors of TB control are complex and wide-ranging [47–49], and the perspective of Health in All Policies (HiAP) emphasizes the need to integrate health considerations into all policies [50]. National TB program in China emphasized the leading role of the government, and required all society to actively participate and provide support [51], which aligns with the principles of HiAP [50].

According to CFIR framework, the inner setting includes features of structural and cultural contexts through which the implementation process will proceed [23]. In this study, inner settings for TCP widely included structural characteristics of PHC sectors, networks and communication between PHC sectors and CDC or TB designated hospitals, implementation climate (tension for change among TB HCWs, compatibility, organizational incentives/rewards, learning climate in PHC sectors) and readiness for implementation (leadership engagement, available resources in PHC sectors). Previous studies pointed out inadequate capacity of HCWs and high staff turnover in PHC sectors were barriers to TCP implementation in PHC sectors [15, 39, 52, 53]. One study pointed out that the lack of coordination and communication among different levels of TB control facilities hindered the implementation of TCP in China [54]. One study reported that the frequent transfer of HCWs led to the disruption of TB care and the dissatisfaction of TB patients [55]. Recent study in Southwest China found that barriers to TB treatment management included insufficient training and poor cross-sectional coordination [39]. This study also observed a high mobility of TB HCWs especially in rural areas. The reasons led to high mobility of trained HCWs included heavy workloads, insufficient work incentives and unreasonable performance review systems. Another key barrier identified in this study was the insufficient cooperation among healthcare institutions within the integrated TB control model, particularly the lack of communication between the PHC sectors and TB-designated hospitals. Besides, this study also revealed that HCWs’ fear of being infected, lack of qualified training program, unreasonable performance review system with insufficient incentives were barriers to TCP implementation in PHC sectors.

The fourth domain pays attention to individuals involved in the intervention and implementation process, who are carriers of cultural, organizational, professional mindsets, norms, interests, and affiliations [22]. This study included TB patients’ and HCWs’ knowledge and beliefs, self-efficacy, stage of change (of utilizing and delivering TCP) and self-identification towards TCP in PHC sectors. A systematic review found that most articles reported factors of TB control in PHC sectors on demographic and social factors of TB patients, including low health awareness, living in areas of middle and high TB burden and stigma related to TB particularly in West China [47]. Another study found that barriers to TB treatment management included low socioeconomic status, poor health literacy and TB-related social stigma at the patient level [20]. In align with another study [45], this study found that HCWs’ TB care knowledge was poor, and highlighted inadequate competency among TB HCWs was a significant barrier to TCP implementation in West China. Recent study reported substantial TB HCWs had job burnout in Chongqing [56]. This study also revealed that although HCWs valued TCP as an effective program, TB patients’ dissatisfaction and resistance to TCP diminished HCWs’ enthusiasm and willingness of TCP implementation in PHC sectors.

The fifth domain is the implementation process, which usually requires active approaches to promote the adoption of the intervention and to achieve individual and organizational level use of the intervention as designed [57]. Development of TCP implementation strategies in PHC sectors in the guideline of BPHS, engaging HCWs within the integrated TB control model and related leaders outside the integrated TB control model, executing TCP according to the requirement in the guideline of BPHS, reflecting and evaluation of TCP implementation were main constructs in this domain. *End TB Strategy* in 2014 recommended that patient-centered care and supervision must be carried out in a context-specific and patient-sensitive manner [3]. Therefore, it is important to involve TB patients and HCWs in the planning of TCP. But this study highlighted that TB patients and HCWs were never involved in the development of TCP implementation, particularly in approaches to TB treatment management, which could explain their low acceptance and satisfaction towards services in West China. Align with previous studies [39, 58], this study found that inappropriate evaluation system diminished HCW’s enthusiasm and willingness to implement TCP in PHC sectors. Though the recommended indicators were used to evaluate HCWs’ performance [51], there was the lack of comprehensive understanding of TCP implementation in PHC sectors. So further study on the evaluation of implementation outcomes is urgent needed.

### Implications

To our knowledge, this is the first study to systematically explore factors (barriers and enablers) of TCP implementation using the CFIR, which provide important evidence to promote TCP in study place and even other regions in the world. Although this study has paid attention to both patients and HCWs in TCP implementation of PHC sectors, research on TB HCWs in designated hospitals and CDC should also be emphasized to explore and evaluate TCP implementation under the integrated TB control model, which need further research in the future.

Strategies to ensure enablers in TCP implementation can be promoted further, such as adapting TCP to local communities and providing technological support to PHC sectors. To address barriers in TCP implementation, this study generated implications for research, policy making and practice. To address major barriers such as TB patients’ non-adherence to TCP and none TB patients’ involvement in the development of TCP, patient-centered approach should be considered in the development and implementation of TCP with sufficient funding support in PHC sectors. Regarding major barriers such as insufficient support from external organizations and relevant departments, this study highlights the need of involving more government institutes to provide financial, network, information and incentive support during the TCP implementation in PHC sectors, and suggests TCP implementation should be integrated into broader policies and strategies such as medical insurance coverage, poverty reduction and education. To improve inner settings for TCP implementation and address major barriers in HCWs’ and TB patients’ knowledge and beliefs towards TCP in PHC sectors, further study on strategies could be conducted to promote better working incentives, establish better performance review and reward system, improve effective communication and collaboration within the integrated TB control model, and strengthen health education for TB patients and professional training for HCWs in PHC sectors. Besides, further study on the evaluation of TCP implementation outcomes in PHC sectors is urgent needed, which may address major barriers such as unreasonable performance assessment system during the TCP implementation process. Notably, though we have provided discussion on possible strategies above, future implementation research with better design is needed to develop specific strategies using CFRI-Expert Recommendations for Implementation Change Matching (CFIR-ERIC) tool [50].

## Conclusions

The implementation of TCP is critical to end TB in China. This study identified factors (barriers and enablers) of the TCP implementation in West China with the guide of the CFIR framework. The main barriers included the lack of patient-centered perspective in TCP, low TB knowledge and beliefs of TCP among HCWs and TB patients, insufficient cooperation within the integrated TB control model, HCWs’ inadequate capacity to deliver TCP and inadequate support from local governments. The possible implementation strategies to overcome identified barriers, including optimizing intervention, adapting patient-centered strategies, providing practice-oriented training, increasing incentives, strengthening communication channel between PHC sectors and TB-designated hospitals, improving cooperation among multiple departments, increasing implementation funding, as well as enhancing TB awareness among the public. Further implementation studies to explore more systematic strategies and translate them into practice are urgent needed.

### Supplementary Information

Additional file 1. The STROBE checklist.

Additional file 2. The COREQ guideline.

Additional file 3. The codebook.

Additional file 4. The interview guide.

## Data Availability

The datasets used and/or analyzed during the current study are available from the corresponding author on reasonable request.

## Abbreviations

TB: Tuberculosis
PHC: Primary healthcare
TCP: TB Control Program
HCWs: Healthcare workers
CFIR: Consolidated Framework for Implementation Research
COVID-19: Coronavirus disease 2019
WHO: World Health Organization
DS-TB: Drug-susceptible TB
MDR/RR-TB: Multidrug-resistant/rifampicin-resistant TB
CDC: Centers for Disease Control
BPHS: Basic public health service
NFI: Normed Fit Index
RFI: Relative Fit Index
IFI: Incremental Fit Index
TLI: Tracker-Lewis Index
CFI: Comparative Fit Index
RMSEA: Root Mean Square Error of Approximation
HiAP: Health in All Policies
