# Supplementary material for: Implementation factors of tuberculosis control program in primary healthcare settings in China: a mixed-methods using the Consolidated Framework for Implementation Research framework
Source: Infect Dis Poverty. 2024 Jul 8;13:52. doi: 10.1186/s40249-024-01222-3 (PMC11229258; doi:10.1186/s40249-024-01222-3)
Supplement: Supplementary file 3 — Additional file 3. The codebook. [file 40249_2024_1222_MOESM3_ESM.docx]

**Codebook**

| **1.      Intervention characteristics---TCP program** |
| --- |
| 1.1 Intervention source |
| 1.1.1 Enablers |
| 1.1.2 Barriers |
| 1.2 Evidence Strength & Quality |
| 1.2.1 Enablers |
| 1.2.2 Barriers |
| 1.3 Adaptability |
| 1.3.1 Enablers |
| 1.3.2 Barriers |
| 1.4 Complexity |
| 1.4.1 Enablers |
| 1.4.2 Barriers |
| 1.5 Cost |
| 1.5.1 Enablers |
| 1.5.2 Barriers |
| **2.      Outer Setting – factors external to the organization delivering TCP** |
| 2.1 Cosmopolitanism |
| 2.1.1 Enablers |
| 2.1.2 Barriers |
| 2.2 External Policy & Incentives |
| 2.2.1 Enablers |
| 2.2.2 Barriers |
| **3.      Inner Setting - Inner Setting for TCP implementation** |
| 3.1 Structural characteristics |
| 3.1.1 Enablers |
| 3.1.2 Barriers |
| 3.2 Networks & Communication |
| 3.2.1 Enablers |
| 3.2.2 Barriers |
| 3.3 Implementation climate (Tension for change, Compatibility, Organizational incentives & rewards, Learning climate) |
| 3.3.1 Enablers |
| 3.3.2 Barriers |
| 3.4 Readiness for Implementation (Leadership engagement, Available resources) |
| 3.4.1 Enablers |
| 3.4.2 Barriers |
| **4.      Individuals - characteristics of the individuals involved in implementation TCP** |
| 4.1 Knowledge and Beliefs about Intervention |
| 4.1.1 Enablers |
| 4.1.2 Barriers |
| 4.2 Self-efficacy |
| 4.2.1 Enablers |
| 4.2.2 Barriers |
| 4.3 Individual State of Change |
| 4.3.1 Enablers |
| 4.3.2 Barriers |
| 4.4 Individual Identification with Organization |
| 4.4.1 Enablers |
| 4.4.2 Barriers |
| **5.      Process – processes of TCP implementation** |
| 5.1 Planning |
| 5.1.1 Enablers |
| 5.1.2 Barriers |
| 5.2 Engaging |
| 5.2.1 Enablers |
| 5.2.2 Barriers |
| 5.3 Executing |
| 5.3.1 Enablers |
| 5.3.2 Barriers |
| 5.4 Reflecting & Evaluating |
| 5.4.1 Enablers |
| 5.4.2 Barriers |

Note: *TCP*=TB control program
