# Supplementary material for: Implementation factors of tuberculosis control program in primary healthcare settings in China: a mixed-methods using the Consolidated Framework for Implementation Research framework
Source: Infect Dis Poverty. 2024 Jul 8;13:52. doi: 10.1186/s40249-024-01222-3 (PMC11229258; doi:10.1186/s40249-024-01222-3)
Supplement: Supplementary file 4 — Additional file 4. The interview guide. [file 40249_2024_1222_MOESM4_ESM.docx]

**Appendix 1-1: Guideline for in-depth interviews with HCWs who implement TCP program in PHC sectors**

I. General situation of HCWs

- How long have you worked in PHC sectors?

- Are you a full-time TB HCW? If not, what kind of work do you have to do at the same time?

- How long have you implemented TCP in PHC sectors?

- What is your education level?

- What is your major background?

- What is your current job title?

II. Implementation status of TCP program in PHC sectors

- What do you think of TCP program in PHC sectors? Why?

- How do you think of the implementation of TCP program in your PHC sectors? Why?

- What do you think of the processes of TCP implementation in your PHC sectors? Why?

- Do you think TCP program adapted to this community? Why?

If not, how to adapt the TCP implementation to this community?

-Do you think you have enough knowledge about TCP program implementation?

If not, what kind of knowledge do you need?

-Do you think you have enough skills to implement TCP program?

If not, what skills do you need?

-Have you attended trainings on TCP implementation? If yes, how are the trainings? Are you motivated to learn more knowledge and skills on better TCP implementation? Why?

-Do you believe you can deliver better TCP implementation? If not, what kind of support do you need?

- How about support from PHC sectors, integrated TB control system, from local health commission?

- Do you think there is any need of cross-sector cooperation? If yes, what kind of cooperation from cross-sectors?

- Do you have any difficulties in TCP implementation?

If yes, what difficulties do you have?

- How about those TB patients who received TCP program? Why?

Do TB patients have feedback on TCP implementation? If yes, how about their feedback?

- Are you satisfied with your work in TCP program? Such as salary, incentives and rewards on TCP implementation?

- Do you have any suggestions for improvement of your work in TCP implementation?

**Appendix 1-2: Guideline for in-depth interviews with leaders related to TCP program implementation in PHC sectors**

I. General situation of the leaders

- How long have you worked in PHC sectors?

- How much do you involve with implementation of TCP in your PHC sectors?

- What is your current job title?

II. Implementation status of TCP program in PHC sectors

- What do you think of TCP program in PHC sectors? Why?

- How do you think of implementation of TCP program in your PHC sectors? Why?

- What do you think of the processes of TCP implementation in your PHC sectors? Why?

- Do you think TCP program adapted to this community? Why?

If not, how to adapt the TCP to your community?

-How do you think of HCWs’ daily work on TCP implementation?

- How do you think of the support for TCP program implementation in your PHC sectors? How about, such as cooperation within integrated TB control model, cross-sector’s cooperation, technical support, policy and incentives, etc.? Are you satisfied with TCP implementation or face any challenges?

- As leaders, what roles do you think you play in TCP implementation?

- Do they have any suggestions for improvement in TCP implementation in your PHC sectors? Do you have any more suggestions?

**Appendix 1-3: Guideline for in-depth interviews with TB patients**

I. General situation of TB patients

- How long have you been treated?

-How do you think of TB?

-TB did any impact on you? If yes, what is/are the main impact(s)?

II. Acceptance of TCP program in PHC sectors

- Do you know about TCP program in PHC sectors? If yes, what do you think of TCP program in PHC sectors? Why?

- What are the main services in TCP program in PHC sectors?

How do you think of those services? Why?

Who supervised your treatment? Do you like he or she? Why?

What are the main approaches to supervise you?

Do you like those approaches? Why?

How do you think of HCWs who provide TCP in PHC sectors? Why?

- What is/are difficulty(ies) you faced during your treatment?
